# Low-Level Laser Irradiation Stimulates Tenocyte Migration with Up-Regulation of Dynamin II Expression

**DOI:** 10.1371/journal.pone.0038235

**Published:** 2012-05-30

**Authors:** Wen-Chung Tsai, Chih-Chin Hsu, Jong-Hwei S. Pang, Miao-Sui Lin, Ying-Hsun Chen, Fang-Chen Liang

**Affiliations:** 1 Department of Physical Medicine and Rehabilitation, Chang Gung Memorial Hospital, Taoyuan County, Taiwan; 2 College of Medicine, Chang Gung University, Taoyuan County, Taiwan; 3 Graduate Institute of Clinical Medical Sciences, Chang Gung University, Taoyuan County, Taiwan; Massachusetts General Hospital - MMS, United States of America

## Abstract

Low-level laser therapy (LLLT) is commonly used to treat sports-related tendinopathy or tendon injury. Tendon healing requires tenocyte migration to the repair site, followed by proliferation and synthesis of the extracellular matrix. This study was designed to determine the effect of laser on tenocyte migration. Furthermore, the correlation between this effect and expression of dynamin 2, a positive regulator of cell motility, was also investigated. Tenocytes intrinsic to rat Achilles tendon were treated with low-level laser (660 nm with energy density at 1.0, 1.5, and 2.0 J/cm^2^). Tenocyte migration was evaluated by an *in vitro* wound healing model and by transwell filter migration assay. The messenger RNA (mRNA) and protein expressions of dynamin 2 were determined by reverse transcription/real-time polymerase chain reaction (real-time PCR) and Western blot analysis respectively. Immunofluorescence staining was used to evaluate the dynamin 2 expression in tenocytes. Tenocytes with or without laser irradiation was treated with dynasore, a dynamin competitor and then underwent transwell filter migration assay. *In vitro* wound model revealed that more tenocytes with laser irradiation migrated across the wound border to the cell-free zone. Transwell filter migration assay confirmed that tenocyte migration was enhanced dose-dependently by laser. Real-time PCR and Western-blot analysis demonstrated that mRNA and protein expressions of dynamin 2 were up-regulated by laser irradiation dose-dependently. Confocal microscopy showed that laser enhanced the expression of dynamin 2 in cytoplasm of tenocytes. The stimulation effect of laser on tenocytes migration was suppressed by dynasore. In conclusion, low-level laser irradiation stimulates tenocyte migration in a process that is mediated by up-regulation of dynamin 2, which can be suppressed by dynasore.

## Introduction

Low-level laser therapy (LLLT) has been used to treat musculoskeletal pain for nearly 3 decades [1]. Clinical applications show its potential of effectiveness in treating soft tissue musculoskeletal injuries, chronic pain, and wound healing [2]. Many studies have revealed the effectiveness of LLLT to treat patients with tendinopathy [3]–[6]. Although the wide spread clinical usage of this physical modality suggests its efficacy, scientific evidence of the effects and underlying molecular mechanisms for tendinopathy treatment remained limited.

In-vivo studies have revealed that LLLT could enhance healing in Achilles tendon by improving collagen fibers organization, preventing oxidative stress as well as reducing fibrosis [7]–[9]. The exact mechanism is still being explored and debated but it is likely that the mechanism is photochemical rather than thermal [10]–[12]. The primary biophysiological effects include regulation of adenosine triphosphate (ATP) [13]–[16], reactive oxygen species (ROS) [17]–[21], and nitric oxide (NO) [22]–[24]. In the regenerative phase of tendon injury, the tenocytes migrate into the repaired site, proliferate actively, and are responsible for the abundant deposition of extracellular matrix (ECM) in the tissue. It was demonstrated that laser irradiation could promote porcine tenocyte proliferation and up-regulation of type I collagen and decorin [25]. Among physical agents, therapeutic ultrasound and electric stimulation have been demonstrated to enhance tenocytes and ligament fibroblast migration respectively [26], [27]. However, to our knowledge, there is no study exploring the effect and underlying molecular mechanism of laser irradiation on tenocyte migration.

Cell migration involves the rearrangement of specific cellular structures, such as the Golgi complex [28], the centrosome [29] and focal adhesions [30]. Dynamins are large molecular weight GTPases∼100 kDa in size and reside at these diverse cellular locations. Along with its well-documented role in endocytosis, dynamin plays a significant role in cell migration [31]. Dynamin 1 is restricted to neuronal cells; dynamin 2 is ubiquitously expressed; and dynamin 3 may be limited to brain, lung and testis. Expression of mutant dynamin 2 or knocking down dynamin 2 by RNAi reduces lamellipodial extension [32]. Besides, injections of anti-dynamin antibodies into amoeba result in a loss of directional migration and a reduction in the rate of uroidal translocation [33].

The purpose of this study is to investigate the effect of laser on tenocyte migration and its correlation with the mRNA and protein expressions of dynamin 2.

## Methods

All the procedures were approved by Chang Gung University Institutional Animal Care and Use Committee before the experiments.

### Primary Culture of Rat Achilles Tenocytes

The Achilles tendons from 16 Sprague-Dawley rats (weighing 200 to 250 gm) were excised. The excised tendon was soaked in povidone-iodine for 3 minutes and washed twice in phosphate-buffered saline (PBS). Each tendon was then cut into small pieces of approximately 1.5–2.0 mm^3^ (6 pieces in total) and these pieces were individually placed in six-well culture plates. After 5 minutes of air-drying for better adherence, 0.5 ml of Dulbecco’s modified Eagle’s medium (DMEM)(HyClone, Logan, Utah, USA), with 10% fetal bovine serum (FBS)(Cansera, Rexdale, Ontario, Canada), 100 U/ml penicillin, and 100 µg/ml streptomycin was added to each well. The explants were then incubated at 37°C in a humidified atmosphere of 5% CO_2_/95% air. After migrating out from the explants, the cells started to grow rapidly and the confluence culture was subcultured by trypsin digestion at a 1∶3 dilution ratio. Tendon cells between passages 2 and 4, with proper growth rate and normal fibroblast-shape, were used in the following experiments.

### Laser Irradiation Procedure

Laser irradiation was carried out with 660 nm laser (Konftec, Megalas-AM-800, New Taipei City, Taiwan) in continuous mode with output power of 50 mW and unit energy density of 0.0032 J/sec-cm^2^. The power of irradiation was uniformly checked with a power meter before and after treatment. The laser beam irradiated the culture plate from above with a distance of 30 cm and covered the area up to 314 cm^2^. The laser beam was emitted perpendicularly and evenly to the culture plates for three groups of wells for periods of 5.2, 7.8, and 10.4 min, respectively. The corresponding energy densities were 1.0, 1.5, and 2.0 J/cm^2^. The irradiation was done on a clean bench through the medium culture at room temperature. The control groups were not subjected to laser irradiation but were removed from the incubator for the same time period as laser treated plates. The cell migration, mRNA expression and protein expression were assessed 24 h after laser irradiation.

### *In vitro* Wound Healing

Tenocytes were grown to confluence on glass coverslips in DMEM with 10% FBS. The confluent cell layer was scraped with a sterile pipette tip to consistently produce a circular cell free zone (1 cm in diameter) on the coverslips. The wound cultures were then incubated at 37°C for 24 hours and the in vitro wound healing was observed under microscope. The control groups were tenocytes without laser treatment, whereas the experimental groups were treated with 2.0 J/cm^2^ laser. During the 24 h incubation in growth medium at 37°C, tenocytes began outgrowth and migrated in to the cell free zone which was considered as the process of in vitro healing. The in vitro wound healing was photographed at 24 h after laser treatment.

### Transwell Filter Migration Assay

Transwell filters (Costar, Cambridge, MA, USA) with 8.0 µm pores were used for the migration assay. Tenocytes with and without laser treatment at various doses (controls *versus* cultures treated with 1.0, 1.5, and 2.0 J/cm^2^ laser) were seeded at a density of 1.2×10^5^ cells per inner chamber of filter. The inner chamber was filled with 250 µl serum-free DMEM and the outer chamber was filled with 600 µl DMEM with 10% FBS. Cells were allowed to migrate for 3 h at 37°C in an atmosphere of 95% air/5% CO2. The cells were stained with Liu’s stain and then washed twice in PBS. Cells on the upper surface of the filter were removed using a cotton swab. The cells on the lower surface of the filter was counted under four random high-power microscopic fields (HPF)(100×)(1.37×10^−4^ cm^2^) per filter and the mean number of migrating cells was calculated for each concentration.

### Reverse Transcription/Real-time Polymerase Chain Reaction (Real-time PCR)

Total RNA was extracted from tendon cells using solution D (1 ml solution D/10^7^ cells). Subsequently, total RNA was extracted with phenol and chloroform:isoamyl alcohol (49∶1) to remove proteins and genomic DNAs. Complementary (c)DNA was synthesized using 1 mg total RNA in a 20 ml volume RT reaction mix containing 0.5 mg of random primers, 0.8 mMdNTP, 0.1MDTT and 1L first strand buffer.). Quantitative real-time PCR was performed using an SYBR Green and MxPro-Mx3000P QPCR machine (Stratagene). Aliquots (20 ng) of cDNA were used for each quantitative PCR, and each reaction was run in triplicate. The following primers were used. GAPDH: 5′-TTCATTGACCTCAACTACAT-3′ (forward) and 5′-GAGGGGCCATCCACAGTCTT-3′ (backward). Dynamin 2: 5′-AGAACGGCAAGTGGAAAC-3′ (forward) and 5′-AGCATAGGCAGCAGGTCA-3′ (backward). Relative gene expressions between experimental groups were determined using MxPro software (Stratagene) and GAPDH was used as an internal control. All real-time PCRs were performed in triplicate, and changes in gene expressions were presented as multiples of increases relative to the untreated controls.

### Western Blot Analysis

Cell extracts were prepared in lysis buffer containing Tris-HCl (pH 7.5), 150 mM NaCl, 1 mM EDTA, 2 mM DTT, 2 mM PMSF and 1% Triton X-100 followed by sonication method. Protein concentration of the cell extracts were determined by Bradford assay (Bio-Rad Laboratories, CA, USA). Samples with identical protein quantities were then separated by 10% sodium dodecyl sulfate polyacrylamide gel electrophoresis, and transferred onto a PVDF membrane. The membrane was incubated at room temperature in blocking solution (1% BSA, 1% goat serum in PBS) for 1 h, followed by 2-h incubation in blocking solution containing an appropriate dilution of primary antibody, eg. anti-tubulin, anti-dynamin 2 (NeoMarks, Fremont, CA, USA). After washing, the membrane was incubated in PBS containing goat anti-mouse IgG conjugated with horseradish peroxidase (Sigma, St. Louis, MO, USA) for 1 h. The membranes were washed and the positive signals developed with enhanced chemiluminescence reagent (Amershan Pharmacia Biotech, Little Chalfont Buckinghamshire, UK).

### Immunofluorescence Staining

Subconfluent tenocytes grown on glass coverslips placed on the bottom of plastic dishes containing growth medium were then treated without or with laser at dosage of 2.0 J/cm^2^. Twenty-four hours after incubation at 37°C in an atmosphere of 95% air/5% CO2., the cells were fixed in 4% paraformaldehyde in PBS (pH 7.5) for 15 min at room temperature. The coverslips were first immersed for 30 min in blocking solution that contained 1% bovine serum albumin (BSA) and 1% goat serum in PBS. After three washings in PBS, the cells were incubated for 1 h with mouse monoclonal antibodies against dynamin 2 (NeoMarks, Fremont, CA, USA), followed by incubation with FITC (flourscein isothiocyanate)-conjugated secondary antibody for 1 h. Finally, DNA was counterstained with propidium iodide (PI) and observed under confocal microscopy.

### Dynasore Treatment

Dynasore, a known dynamin inhibitor, was added at 3 µg/ml to tenocytes without or with 2.0 J/cm^2^ laser treatment. After 24 h of incubation, transwell filter migration assay was performed to evaluate the effect of dynasore treatment alone as well as the combined effect of dynasore and laser treatment on tenocytes migration.

### Statistical Analysis

All data was expressed as mean±SEM. Comparisons between the results of tenocyte migration and spreading assays of the laser-treated and control cells was performed using Kruskal-Wallis test. A Mann-Whitney test was used to identify where the difference occurred. The level of statistical significance is set at a *P* value of 0.05.

## Results

**Figure 1:**
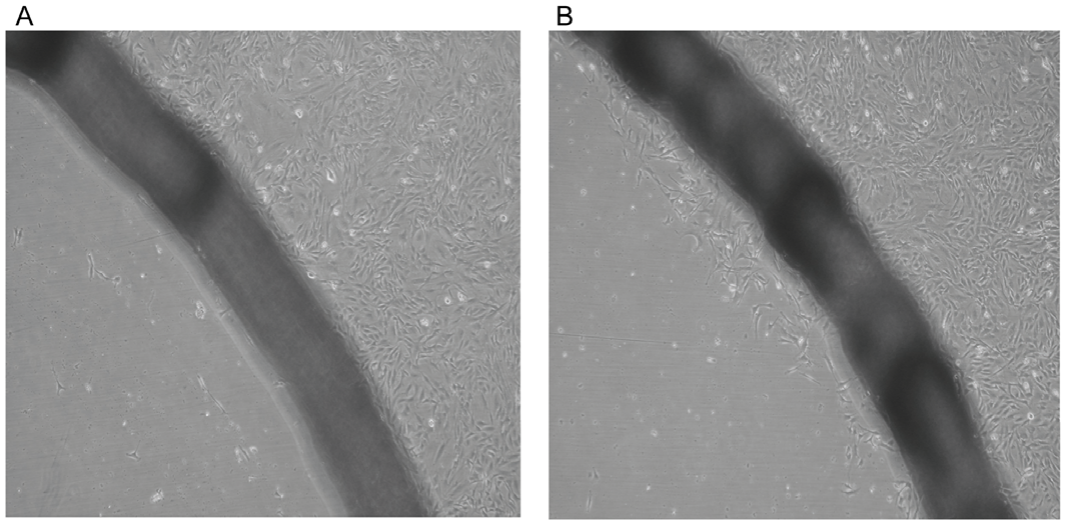
*In vitro* wound healing model revealed that few control cells (without laser treatment) migrated cross the wound border to enter the cell-free zone (A). Laser-treated group (B) revealed that more tenocytes migrated cross the wound border to enter the cell-free zone (200X).

The *in vitro* wound healing model revealed that more tenocytes in the laser-treated groups have migrated cross the wound border and entered the cell-free zone. However, only few control tenocytes were observed near the wound border (Figure 1). This result indicated that laser irradiation could enhance the in vitro wound healing.

**Figure 2:**
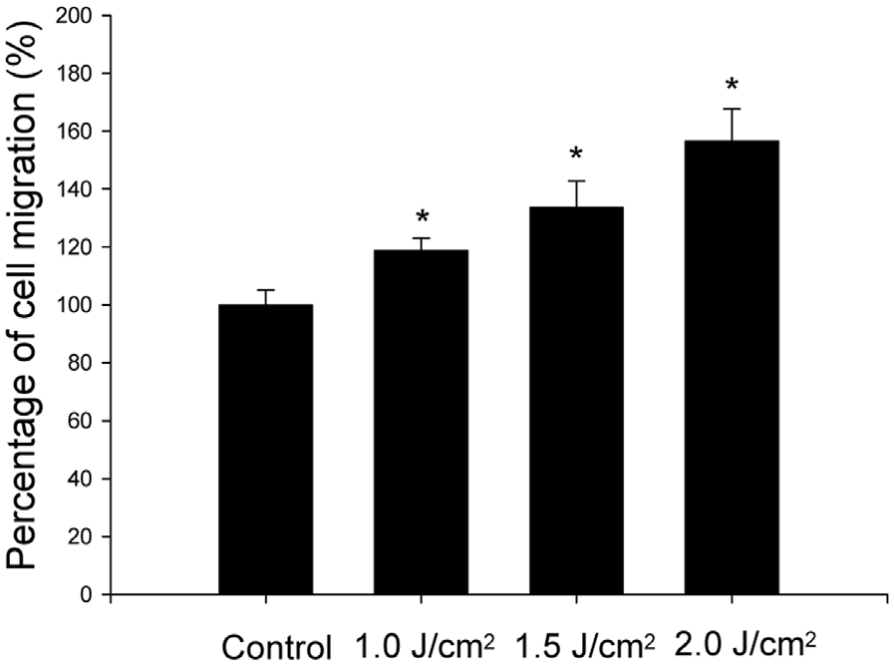
Transwell filter migration assay revealed that laser stimulated tenocytes migration *in vitro* (*indicates *p*<0.05 between laser-treated and control tenocytes).

The results of transwell filter migration assay demonstrated that laser significantly enhanced the tenocyte migration in a dose-dependent manner (Figure 2). The relative percentage of laser-treated tenocytes that migrated through the filters to the control tenocytes were 118.8±4.6%, 133.7±9.0%, and 156.5±11.1% for tenocytes treated with 1.0 J/cm^2^, 1.5 J/cm^2^ and 2.0 J/cm^2^ laser respectively (*p* = 0.006). There are statistically significant differences of cell migration between the control and tenocytes treated with 0.1 J/cm^2^ laser (*p* = 0.014), tenocytes treated with 1.0 J/cm^2^ and 1.5 J/cm^2^, (*p* = 0.043), as well as tenocytes treated with 1.5 J/cm^2^ and 2.0 J/cm^2^ (*p* = 0.021).

**Figure 3:**
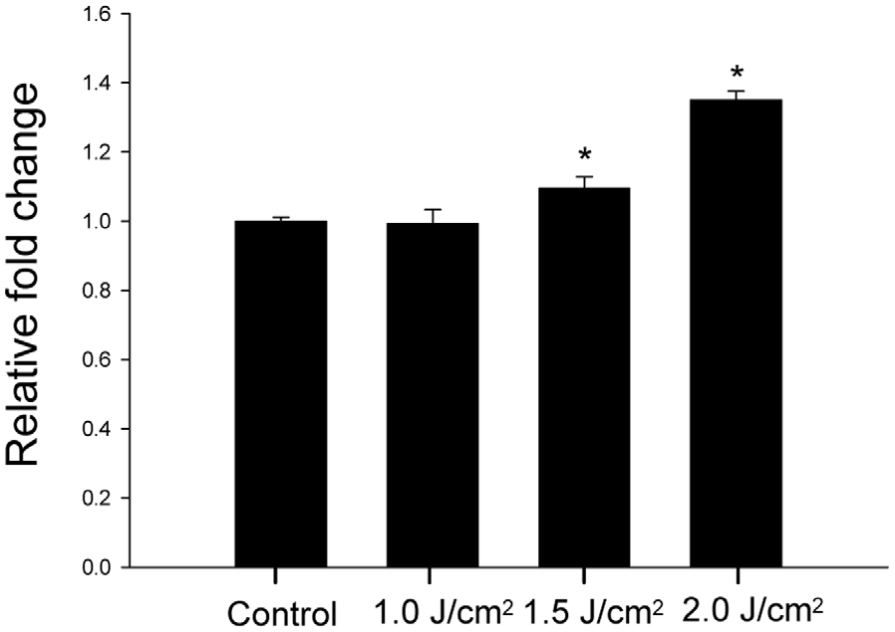
Real-time PCR revealed that the expression of dynamin 2 was up-regulated by laser (*indicates *p*<0.05 between laser-treated and control tenocytes).

The result of real-time PCR demonstrated that the mRNA expression of dynamin 2 in tenocytes was up-regulated by laser dose-dependently (1.02±0.02, 1.14±0.02, 1.35±0.01 relative folds for tenocytes treated with 1.0 J/cm^2^, 1.5 J/cm^2^ and 2.0 J/cm^2^ laser respectively, *p* = 0.029) (Figure 3). There are statistically significant differences of mRNA expression between tenocytes treated with 1.0 J/cm^2^ and 1.5 J/cm^2^ (*p*≤0.005) as well as tenocytes treated with 1.5 J/cm^2^ and 2.0 J/cm^2^ (*p*≤0.005).

**Figure 4:**
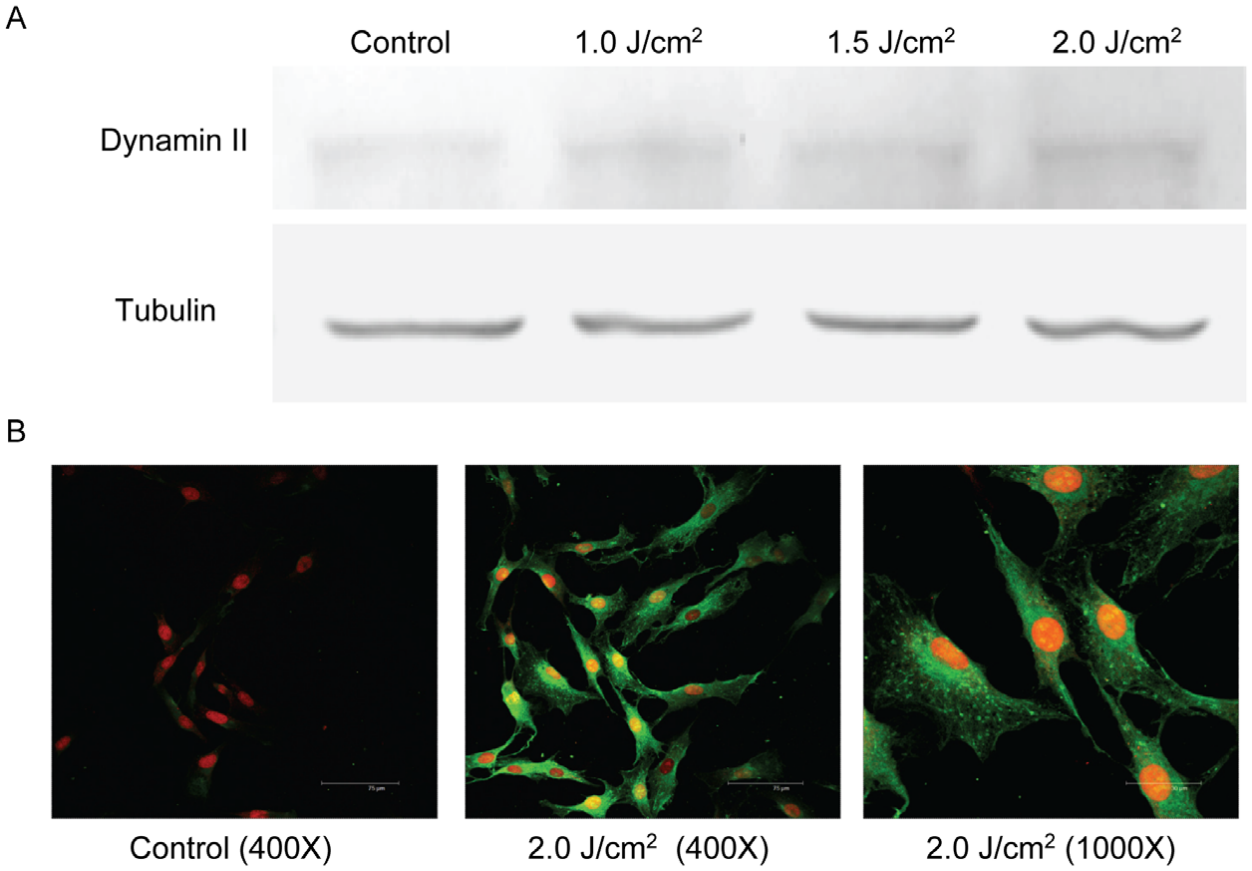
The expression of dynamin 2 in tenocytes. Western blot analysis revealed that dynamin 2 was up-regulated by laser treatment. The tubulin (as internal control) and dynamin 2 were identified at 57 kDa and 100 kDa respectively. (A) Immnofluorescence staining revealed the significantly increased dynamin 2 expression in cytoplasm as indicated by fluorescent-green stain.

Protein expression of dynamin 2 was also dose-dependently up-regulated by laser treatment, which was compatible with the results of real-time PCR (Figure 4A). The cellular protein expression of dynamin 2 was further examined by immunostaining of dynamin 2 and observed under fluorescent confocal microscopic. As revealed by Figure 4B, the cellular expression of dynamin 2 in control tenocytes was barely detected (i.e., fluorescent-green staining). However, in laser-treated tenocytes, the dynamin 2 expression was markedly increased in the cytoplasm. The cellular distribution of dynamin 2 in tenocytes was similar to the pattern reported for cells with high migratory potential in previous study. The significantly up-regulated expression of dynamin 2 was correlated well with the increase of numerous large lamellipodia protrusion [34]. These findings were compatible with the results of Western-blot analysis which confirmed that the protein expression of dynamin 2 in tenocytes could be significantly up-regulated after laser treatment.

**Figure 5:**
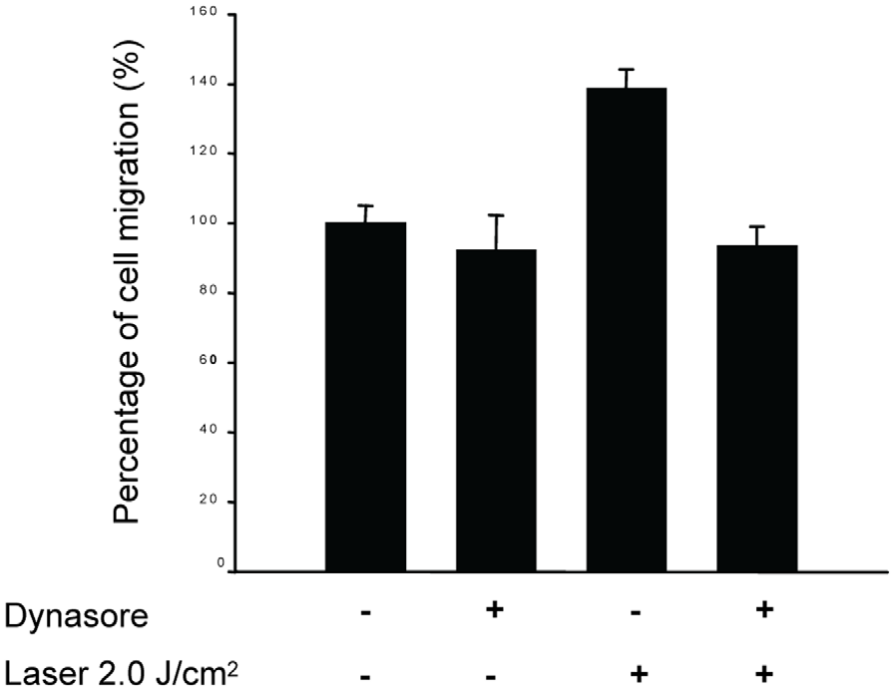
Dynasore inhibited the stimulation of tenocyte migration by low-level laser irradiation. Laser can not stimulate migration of tenocytes pre-treated with dynasore (“+”: with dynasore or laser treatment; “−”: without dynasore or laser treatment).

To further investigate the role of dynamin 2 in the laser-induced migration of tenocytes, we pre-treated the cells with dynasore and study its effect on cell migration. Dynasore, a non-competitive inhibitor of dynamin GTPase activity, locks dynamin in a GTP-bound state likely through an association with the GTPase domain. The results of transwell filter migration assay revealed that the migration of tenocytes without laser treatment was only mildly suppressed (93.7±11.8%) by dynasore (Figure 5). But it was not statistically significant. In contrast, the stimulation of tenocytes migration by 2.0 J/cm^2^ laser (140.0±10.9%) was significantly suppressed by dynasore treatment (93.6±2.7%) (*p* = 0.008). The inhibition of dynamin 2 activity by dynasore could decrease the laser-induced tenocyte migration, which indicates the critical role of dynamin 2 in this migratory process.

## Discussion

Tendon structure consists mainly of dense collagen arranged in a linear fashion and the basic cellular component, i.e. tenocytes (tendon cells; fibroblasts). Tenocytes, appearing as a stellate cell in cross-sections and in rows in longitudinal section, are the source of collagen production, protein mediators of repair, and matrix proteoglycans [35], [36]. For an injured tendon, the healing process can be divided into three overlapping phases: (1) inflammation; (2) regeneration; and (3) remodeling and maturation [36]. In the regenerative phase of tendon injury, tenocytes migrate into the repaired site, proliferate actively, and are responsible for the abundant deposition of ECM in the tissue. Tenocyte migration is fundamental to the healing process of an injured tendon. It was demonstrated that laser could enhance fibroblasts attachment to root surfaces, which implied that laser might have a positive effect on fibroblast motility [37]. It was also demonstrated that laser phototherapy can stimulate migration and fiber sprouting of neuronal cells aggregates [38], [39]. To our knowledge, this study was the first to demonstrate that laser, in a dose-dependent manner, can enhance tenocytes migration, which is associated with up-regulations of mRNA and protein expressions of dynamin 2.

Cell migration is led by pseudopodia such as lamellipodia or filopodia, plasma membrane protrusive structure enriched with F-actin. The large GTPase dynamin, long known for its role in endocytosis, has most recently been implicated as a facilitator of cell migration and invasion [31], [40]. Recent reports link dynamin to the cycle of membrane expansion and retraction essential for cell motility. It indicates that dynamin might promote efficient cell migration by participating in lamellipodial extension. Its role in actin polymerization, membrane deformation and vesiculation, and focal adhesion dynamics are all important for this process.

It is reported that that dynamin 2 interacts and co-localizes with focal adhesion kinase (FAK) at focal adhesions [41]. It was reported that cells expressing the dynamin 2 mutant have reduced rates of migration into a wound and exhibit a characteristic drag of the uropod, which suggests an inappropriate persistence of focal adhesion attachment.

Dynasore, a membrane permeable small molecule, decreases endocytosis, cell spreading, and cell attachment and is a non-competitive inhibitor of dynamin [42]. It has been widely used to investigate dynamin-dependent endocytosis [43] Dynasore strongly destabilizes F-actin both in vitro and in vivo. Application of dynasore led to reduction of lamellipodia formation and inhibition of cellular invasion by destabilizing actin filaments [44]. It has been demonstrated that dynasore led to dissociation of dynamin 2 from F-actin, and reduction of dynamin 2-positive dots on F-actin [44]. Besides, dynasore also interfered the interaction of dynamin 2 and cortactin [44]. It was demonstrated that dynasore provides a means to rapidly block the formation of clathrin-coated vesicles, with the initial effects being observable within seconds and a more complete halt in coated-pit dynamics occurring within minutes [42]. The results of this study revealed that dynasore could inhibit laser stimulation of tenocyte migration. These findings verify the role of dynamin 2 in mediating laser stimulation of tenocyte migration.

The laser dose used in this study was between 1.0 and 2.0 J/cm^2^ which is within the recommend dose range (0.1 to 3.0 J/cm^2^) to treat tendinopathy [45]. The upper limit of laser dose in our study was 2.0 J/cm^2^ because higher doses (≥2.5 J/cm^2^) would decrease cell viability and inhibit proliferation of tenocytes in this experimental setting. This finding indicated a biphasic dose response in cell viability of tenocytes treated with low level laser. A decrease in cell viability may have a negative impact on cellular behaviors including cell proliferation and motility. This finding was similar to the report that laser increases cell proliferation of Achilles tendon fibroblasts, with the optimal dose being 2 J/cm^2^. Higher doses of laser stimulation (3 J/cm^2^) do not enhance cell proliferation on Achilles tendon fibroblasts [25]. The experimental biphasic dose response in cell culture study using tendon fibroblasts may provide an explanation for the similar observation that low level laser may have better effect on promoting the tissue healing compared to high level laser [46].

There are some limitations for this study. First, the results of this study were investigated after 24 h of laser exposure. It is uncertain as to the accumulated effects of repeated laser treatments for a longer period of time on tenocytes, which is a common practice to treat patient with tendinopathy or tendon injury. Second, it should be cautious when extrapolating these *in vitro* results to *in vivo* condition. Therefore, further animal studies are mandatory to verify the findings of this study. However, a thorough understanding of the dose effects and molecular mechanism of laser on tenocytes migration do provide laboratory-based evidence to support the use of laser to treat tendinopathy or tendon injury.

In conclusion, laser irradiation can enhance tenocyte migration with up-regulation of mRNA and protein expressions of dynamin 2. This stimulatory effect can be counteracted by dynasore treatment. These findings provide novel molecular mechanism accounting for LLLT for treating tendinopathy, or tendon injury.
